# *Melipona quadrifasciata* Geopropolis Extract as a Modulator of Inflammation and Pro-Regenerative Responses in Human Macrophages

**DOI:** 10.3390/ijms27073229

**Published:** 2026-04-02

**Authors:** Luiza Naemi Koga Zapotoski, Maria Carolina de Oliveira Ribeiro, Marcelo José Pena Ferreira, Denise V. Tambourgi, Paula Cristiane Pohl

**Affiliations:** 1 Immunochemistry Laboratory, Butantan Institute, São Paulo 05503-900, SP, Brazil; l.zapotoski.proppg@proppg.butantan.gov.br (L.N.K.Z.); denise.tambourgi@butantan.gov.br (D.V.T.); 2 Department of Bioprocess and Biotechnology Engineering, Federal Technology University of Paraná, Ponta Grossa 84016-210, PR, Brazil; carolina@utfpr.edu.br; 3 Department of Botany, Institute of Biosciences, University of São Paulo, São Paulo 05508-090, SP, Brazil; marcelopena@ib.usp.br

**Keywords:** *Melipona quadrifasciata*, geopropolis, macrophages, anti-inflammatory, pro-regenerative

## Abstract

Geopropolis, a complex natural product composed of propolis, wax, plant resins, and soil produced by Meliponine (stingless) bees, has traditionally been used for its therapeutic properties. Its chemically diverse composition and broad biological activities have recently attracted growing scientific interest. In this study, we characterized the physicochemical and immunomodulatory properties of a hydroalcoholic extract of geopropolis (HEG) from *Melipona quadrifasciata* (Mandaçaia). Physicochemical characteristics were determined by measuring moisture, ash, and wax content, and its bioactive constituents were identified by GC–MS. THP-1-derived macrophages were exposed to increasing HEG concentrations to assess cytotoxicity, and two sublethal doses were selected for immunomodulatory assays with or without LPS stimulation. Cytokine and chemokine secretion were quantified by CBA, and the expression of key immunoregulatory and angiogenic genes was evaluated by RT-qPCR. Chemical profiling revealed a high wax content and a predominance of di- and triterpenoids, largely derived from coniferous sources. In mccrophages stimulated with LPS, HEG at 31.25 and 62.50 µg/mL significantly reduced the secretion of pro-inflammatory mediators (IL-6, CCL2, CCL5, CXCL9, and CXCL10) while preserving cell viability. In unstimulated macrophages, HEG upregulated the expression of genes *VEGFA* and *TGFB1* as well as the protein CXCL8, all of them associated with angiogenesis and tissue repair. These findings demonstrate that *M. quadrifasciata* geopropolis extract modulates macrophage activity, promoting a shift toward a reparative phenotype that integrates inflammatory resolution with pro-healing effects. These results underscore its pharmacological potential as a terpenoid-rich natural product with complementary anti-inflammatory and regenerative activities.

## 1. Introduction

Meliponini bees, also known as stingless and native bees, are distributed predominantly across tropical and subtropical ecosystems. Current taxonomic studies have identified over 400 distinct species worldwide, with approximately 300 species occurring in Brazil [1]. These bees play a crucial role in ecosystems as primary pollinators of plants, contributing to biodiversity maintenance through cross-pollination and supporting the reproduction of native plant species [2]. Furthermore, their economic significance is considerable, especially within meliponiculture and in the food sector, where they contribute to the production of propolis, honey, and royal jelly.

Geopropolis is an intrinsic product of meliponinis, primarily used to ensure the structural integrity and waterproofing of their hives. It is produced by mixing beeswax with resins collected from trees, leaves, and flowers. Its composition differs from that of propolis by incorporating earth or clay, which gives rise to the prefix “geo” [3] and often results in the material being discarded in meliponaries. Despite this, geopropolis has attracted scientific interest because of its chemical and biological properties, highlighting its notable therapeutic potential. These properties have long been recognized by Indigenous communities, who have traditionally used geopropolis in their medicinal and cultural practices [4,5,6].

The chemical composition of geopropolis exhibits significant variability influenced by environmental factors, such as climate, geographical region, and seasonal changes, primarily due to the diversity of local flora [3,7]. Its predominant constituents include propolis, waxes, resins, and pollen, along with the distinctive presence of soil or clay as an inorganic fraction, which contributes additional properties derived from minerals and other soil components [8]. The organic fraction is largely composed of phenols, flavonoids, and terpenoids [9,10,11], compounds that have been associated with a variety of biological activities, such as antibacterial [10,12], antiviral [13], and antioxidant effects [14,15].

Recent studies have also investigated the immunomodulatory properties of geopropolis. Franchin and coworkers demonstrated that hydroalcoholic extract of geopropolis produced by *Melipona scutellaris*, a highly enriched sample of phenolic compounds, exerted antinociceptive and anti-inflammatory effects in a mouse model of acetic acid- and formalin-induced abdominal constrictions. These effects were associated with a reduced release of IL-1β and TNF-α, suggesting downstream inhibition of prostanoid production [16]. In a complementary study, the anti-inflammatory potential of geopropolis from *Melipona fasciculata* was investigated, focusing on its ability to inhibit hyaluronidase activity in HL-60 cells (human promyeloblasts of leukemia). This geopropolis extract, rich in sugars of the p-coumaroyl class and terpenes, reduced hyaluronic acid degradation, reinforcing its anti-inflammatory profile [10]. More recently, the effects of *M. fasciculata* geopropolis, composed by carbohydrates, triterpenes, anacardic acid, alkylresorcinols, and sugar alcohols, were investigated on human peripheral blood mononuclear cells; the authors reported reductions in IL-1β, IL-6, and TNF-α levels. Notably, an enhanced anti-inflammatory response was observed when the extract fraction was used in combination with the anti-inflammatory dexamethasone [17].

Macrophages are specialized phagocytic cells widely distributed throughout organs and connective tissues. They play a pivotal role in the innate immune system, contributing to inflammatory processes and the regulation of homeostasis. They act as frontline defenders by ingesting and phagocytizing microorganisms, pathogens, and foreign particles. When a disturbance in the cellular microenvironment occurs, macrophages are among the first immune cells to be activated, producing cytokines and inflammatory mediators that coordinate the recruitment and activation of additional immune cells, thereby enhancing the immune response [18].

Macrophage heterogeneity is a critical factor that influences gene expression and functional responses to various stimuli. Upon activation, these cells can polarize into distinct phenotypes, exhibiting either a pro-inflammatory profile, commonly referred to as classically activated macrophages (M1), or an anti-inflammatory profile, known as alternatively activated macrophages (M2) [19,20]. This polarization is defined by distinct cytokine profiles and differential expression of surface markers [21]. Among the principal mediators involved in macrophage polarization, the most extensively studied include interleukins (IL-1β, IL-4, IL-6, IL-8, IL-10, IL-12), tumor necrosis factor (TNF-α), interferons (IFN-α, IFN-γ), monocyte chemoattractant proteins (MCP-1, MCP-3), transforming growth factor-β (TGF-β), and vascular endothelial growth factor A (VEGFA) [18,22].

Given their crucial role in mediating inflammatory responses and regulating immune processes, macrophages are central players in both physiological and pathological conditions. This importance is particularly highlighted by the global burden of inflammatory disorders, which remain a major challenge for pharmaceutical and medical research. Currently available anti-inflammatory therapies are often constrained by limited efficacy, adverse effects, or long-term toxicity, underscoring the urgent need for novel and safer therapeutic alternatives [23]. In this context, natural products, including geopropolis, have emerged as promising sources of bioactive compounds with immunomodulatory potential.

Motivated by these considerations, the present study aims to characterize the physicochemical properties and immunomodulatory activity of the hydroalcoholic extract of geopropolis (HEG) obtained from the *Meliponaquadrifasciata* bee (known as Mandaçaia) using a well-established human macrophage model (THP-1-derived macrophages).

## 2. Results

### 2.1. Identification of Physicochemical Properties and Compounds

The results of physicochemical analysis of HEG are shown in Table 1. The sample contained 51.81% wax, 4.52% water, and 50.67% ash. Gas chromatography–mass spectrometry (GC-MS) identified 29 compounds in HEG. These included diterpenoids (83.4%), triterpenoids (4.04%), fatty acids (1.7%), and sugars (0.48%) as presented in Table 2.

Lipopolysaccharide (LPS), a major component of the outer membrane of Gram-negative bacteria, is responsible for their endotoxin activity. Because geopropolis is a natural hive-derived product, endotoxin quantification was performed to exclude potential bacterial contamination that could interfere with macrophage activation assays. According to the Brazilian Health Regulatory Agency [24], the bacterial endotoxin test is routinely used as a quality control measure to detect and quantify endotoxins in test samples. For biological assays, the acceptable basal limit varies according to the application, being roughly 0.1 EU/mL based on observed adverse effects such as reduced cell proliferation [25]. The endotoxin content detected in HEG was <0.0083 EU/µg.

### 2.2. Cytotoxicity of HEG on Macrophages

**Figure 1:**
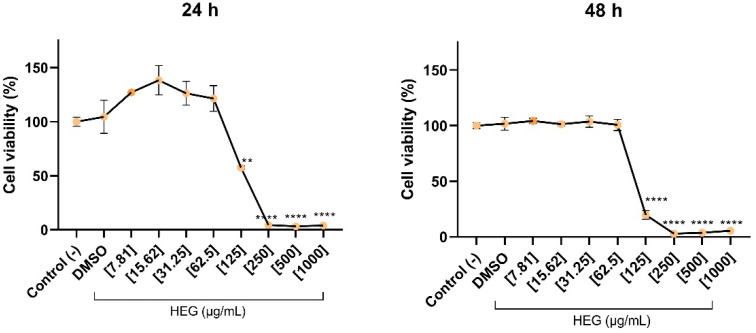
Cytotoxic effects of HEG on macrophages. THP-1-differentiated macrophages (2.5 × 10^4^ cells/well) were treated with HEG at concentrations from 7.81 to 1000 µg/mL. Cells not treated with HEG or with the highest concentration of vehicle DMSO were used as controls. After 24 and 48 h of incubation, cells were treated with MTT to assess viability. Statistical analysis was performed by one-way ANOVA, followed by Dunnett’s post-test comparative to the negative control. (**) *p* < 0.01 and (****) *p* < 0.0001.

To investigate the effects of HEG on macrophages, we first evaluated its impact on cell viability. HEG concentrations above 125 µg/mL markedly reduced macrophage viability, decreasing metabolic activity by more than 50% (Figure 1). In contrast, lower concentrations (7.81 to 62.50 µg/mL) did not cause significant changes in cell viability over the evaluated periods.

Cytokine production was also quantified in culture supernatants to exclude potential pro-inflammatory effects of HEG. IL-6 levels were not significantly modulated, and IL-10 modulation was observed only at lower concentrations. In contrast, significant increases in IL-1β and TNF-α were observed only at higher HEG concentrations, which were associated with reduced cell viability (Figure S1).

Based on these findings, concentrations of 31.25 µg/mL and 62.5 µg/mL were selected for subsequent immunomodulatory assays as they did not significantly reduce cell viability or induce inflammatory responses at either 24 or 48 h.

### 2.3. Immunomodulatory Actions of HEG on Macrophages

**Figure 2:**
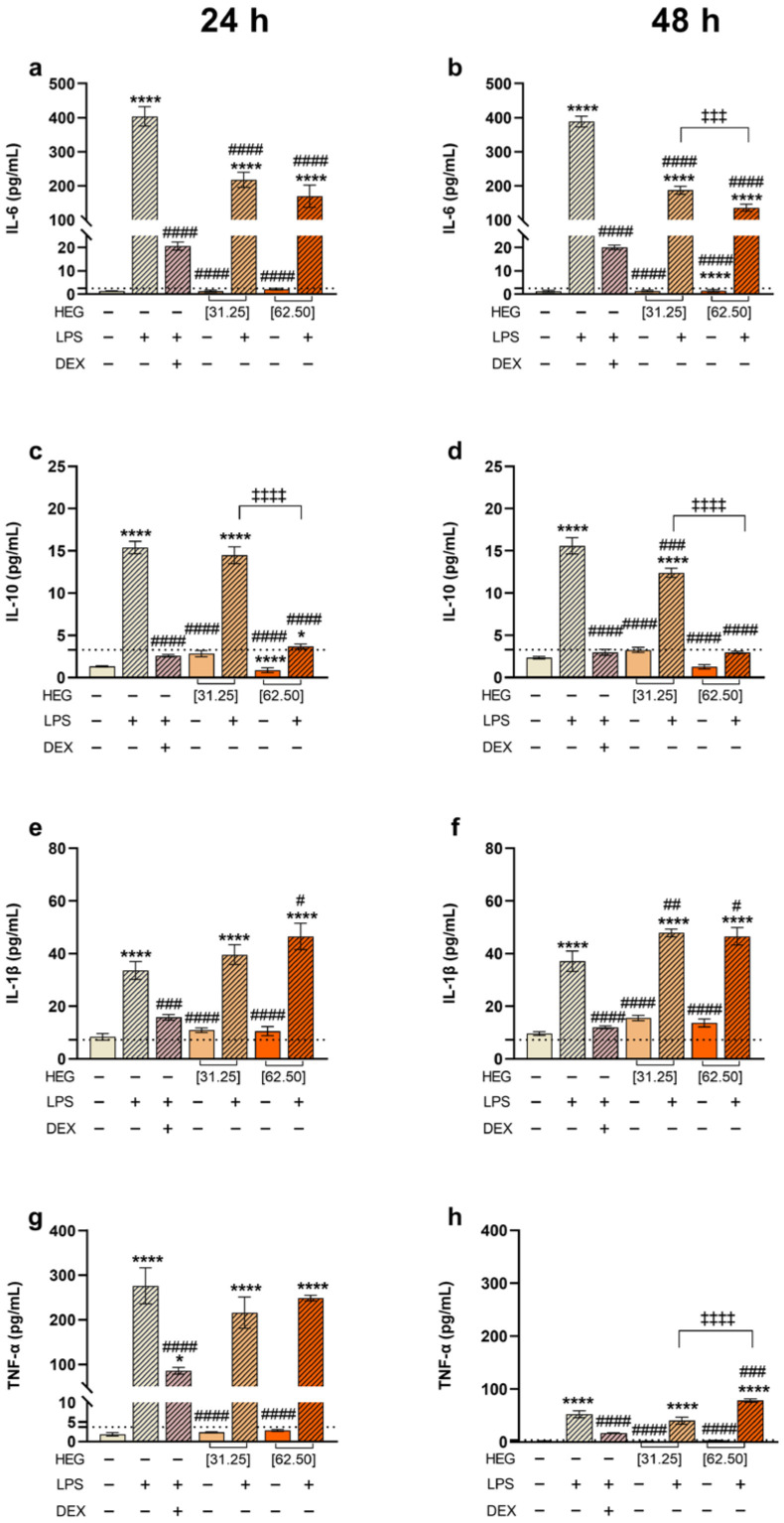
Cytokine levels of macrophages treated with LPS and HEG. THP-1-differentiated macrophages (2.5 × 10^4^ cells/well) were stimulated with LPS (10 ng/mL) or left unstimulated for 1 h and then treated with HEG at 31.25 and 62.5 µg/mL. As controls, cells were maintained in culture medium (negative control), in LPS (positive control) or stimulated with LPS for 1 h and then treated with dexamethasone (DEX, 1 µM). After 24 and 48 h of incubation, the production of IL-6 (**a**,**b**), IL-10 (**c**,**d**), IL-1β (**e**,**f**) and TNF-α (**g**,**h**) in the supernatants of the cultures was determined by CBA. The results represent two separate experiments performed in triplicate and are expressed as the mean of the concentrations of the molecules ± SEM. Statistical analysis was performed by one-way ANOVA, followed by Dunnett’s post-test. Comparative to the negative control (*) *p* < 0.5, and (****) *p* < 0.0001. Comparative to positive control (LPS) (#) *p* < 0.5, (##) *p* < 0.01, (###) *p* < 0.001 and (####) *p* < 0.0001. Comparative between concentrations (‡‡‡) *p* < 0.001 and (‡‡‡‡) *p* < 0.0001. The dotted line represents the limit of detection for each analyte.

To assess the anti-inflammatory potential of HEG, cytokine production was quantified in THP-1 macrophages under inflammatory conditions. As expected, LPS stimulation strongly increased the release of pro-inflammatory cytokines compared with unstimulated macrophages, which produced only minimal levels of these mediators. Additionally, cells exposed to HEG in the absence of LPS displayed cytokine levels comparable to the negative control, indicating that HEG did not induce a pro-inflammatory response. As a reference, dexamethasone, an anti-inflammatory drug, markedly reduced the secretion of all cytokines analyzed (Figure 2).

In contrast, when macrophages were treated with HEG after LPS-stimulation, a significant reduction in IL-6 levels was observed compared with the positive control (LPS+), with a clear dose-dependent difference at 48 h (Figure 2a,b). Interleukin-10 (IL-10), an anti-inflammatory cytokine that suppresses pro-inflammatory mediators’ expression, was also increased in LPS-stimulated macrophages. However, cells treated with HEG after LPS stimulation exhibited a significant reduction in IL-10 production at both 24 and 48 h compared with the positive control. This effect was more pronounced at 62.50 µg/mL and resembled the response observed in dexamethasone-treated cells (Figure 2c,d).

For IL-1β, statistical differences were also assessed between LPS-stimulated cells and subsequently treated with HEG and the positive control (LPS+). At 24 h, a slight difference was observed only for the 62.50 µg/mL HEG. At 48 h, both concentrations of HEG produced a small but statistically significant increase compared with the positive control (Figure 2e,f). TNF-α levels were higher at 24 h than 48 h overall. At 24 h, neither HEG concentration produced significant changes after LPS stimulation. However, at 48 h, a slight but significant increase in TNF-α was observed at 62.50 µg/mL, while the lower dose showed no difference from the positive control (Figure 2g,h). Consistent with these findings, cell viability remained above 80% in all groups (Figure S2).

### 2.4. Human Chemokine Response to LPS Stimulation

**Figure 3:**
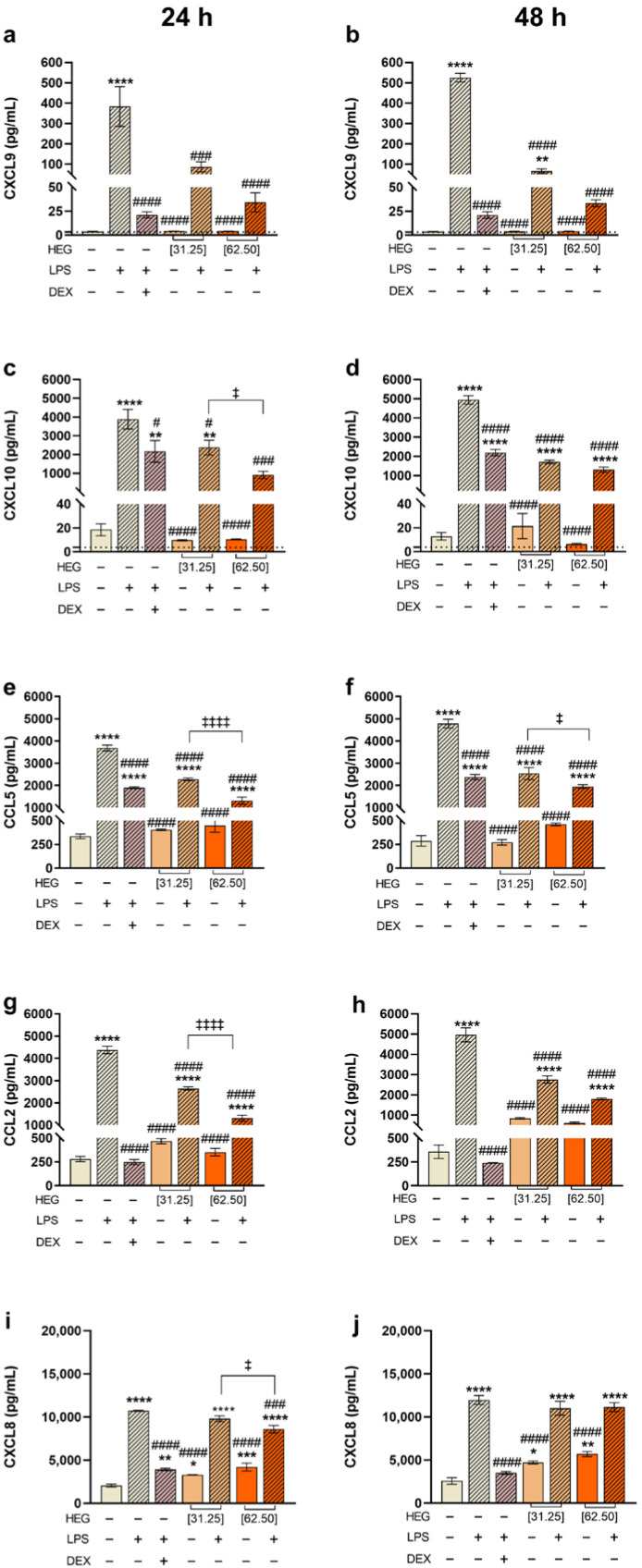
Chemokine levels of macrophages treated with LPS and HEG. THP-1-differentiated macrophages (2.5 × 10^4^ cells/well) were stimulated with LPS (10 ng/mL) or left unstimulated for 1 h and then treated with HEG at 31.25 and 62.5 µg/mL. As controls, cells were maintained in culture medium (negative control), in LPS (positive control) or stimulated with LPS for 1 h and then treated with dexamethasone (DEX, 1 µM). After 24 and 48 h of incubation, the production of CXCL9 (**a**,**b**), CXCL10 (**c**,**d**), CCL5 (**e**,**f**), CCL2 (**g**,**h**) and CXCL8 (**i**,**j**) in the supernatants of the cultures was determined by CBA. The results represent two separate experiments performed in triplicate and are expressed as the mean of the concentrations of the molecules ± SEM. Statistical analysis was performed by one-way ANOVA, followed by Dunnett’s post-test. Comparative to the negative control (*) *p* < 0.5, (**) *p* < 0.01, (***) *p* < 0.001 and (****) *p* < 0.0001. Comparative to positive control (LPS) (#) *p* < 0.5, (###) *p* < 0.001 and (####) *p* < 0.0001. Comparative between concentrations (‡) *p* < 0.5 and (‡‡‡‡) *p* < 0.0001. The dotted line represents the limit of detection for each analyte.

Chemokine concentration in the cell supernatant revealed a similar modulation pattern for CXCL9 (MIG), CXCL10 (IP-10), and CCL5 (RANTES). Macrophages treated with HEG alone did not show increased levels of these chemokines compared with the negative control. In contrast, LPS stimulation markedly increased their production at both times analyzed. Notably, HEG treatment following LPS stimulation produced a significant, dose-dependent reduction in chemokine levels compared with the positive control (LPS+), with the strongest inhibition observed at 62.50 µg/mL, similar to the effect of dexamethasone. This modulation was not influenced by the incubation time (Figure 3a–f). HEG alone did not modulate CCL2 (MCP-1) production compared with the negative control. However, relative to the positive control (LPS+), both concentrations of HEG significantly downregulated CCL2 production at both time points, with the most pronounced inhibition observed at 62.50 µg/mL after 24 h (Figure 3g,h). CXCL8 (IL-8) exhibited the greatest degree of modulation. Treatment with HEG alone increased CXCL8 production relative to the negative control. LPS strongly induced its expression; however, when HEG was added to LPS-stimulated cells, a significant reduction in CXCL8 production was observed only at 62.50 µg/mL after 24 h, with no significant effects at the lower dose or at 48 h (Figure 3i,j).

### 2.5. Relative Gene Expression Analysis

**Figure 4:**
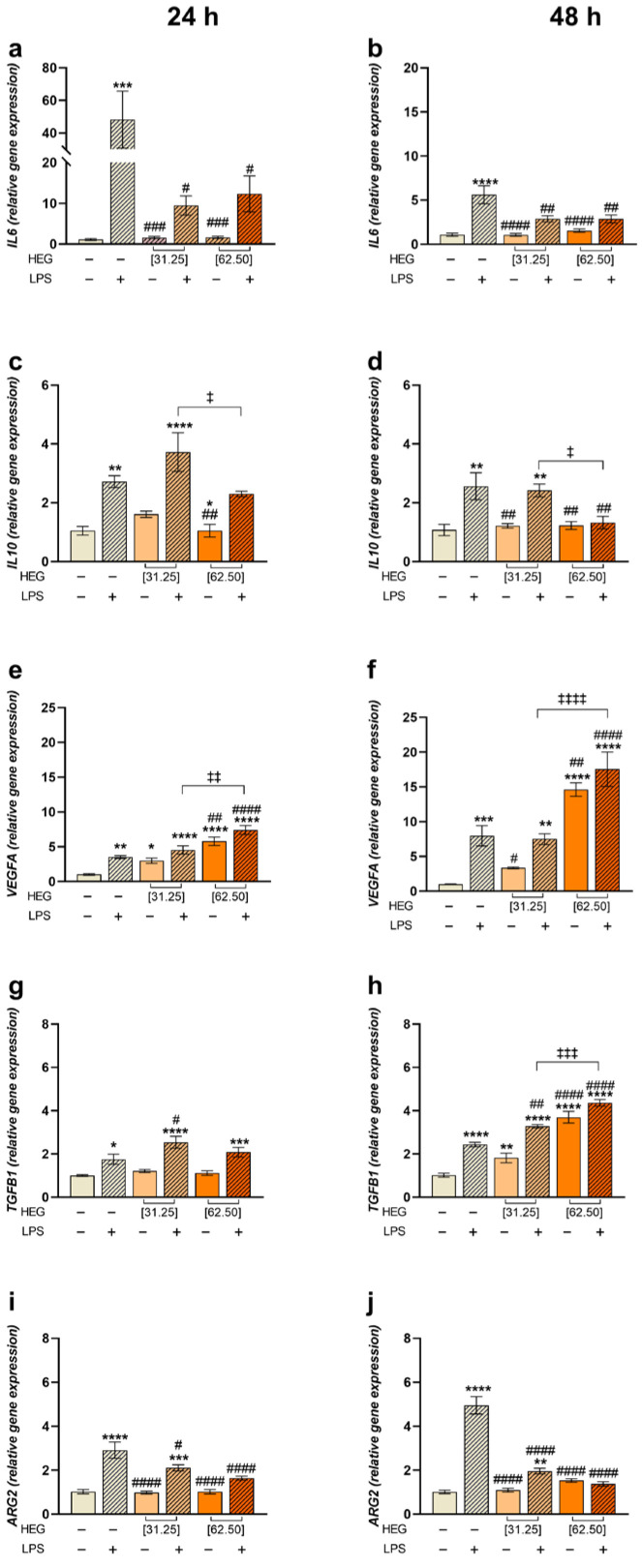
Gene expression analysis in macrophages treated with HEG. THP-1-differentiated macrophages (2.5 × 10^4^ cells/well) were stimulated with LPS (10 ng/mL) or left unstimulated for 1 h and then treated with HEG at 31.25 and 62.5 µg/mL. As controls, cells were maintained in culture medium (negative control) or stimulated with LPS alone (positive control). After 24 and 48 h of incubation, RNA was extracted, and relative mRNA quantification of *IL6* (**a**,**b**), *IL10* (**c**,**d**), *VEGFA* (**e**,**f**), *TGFB1* (**g**,**h**) and *ARG2* (**i**,**j**) was performed using RT-qPCR. The results represent two separate experiments performed in triplicate and are expressed as the mean ± SEM normalized to *GAPDH* and *RPL13A* as endogenous controls. Statistical analysis was performed by one-way ANOVA, followed by Dunnett’s post-test. Comparative to the negative control (*) *p* < 0.5, (**) *p* < 0.01, (***) *p* < 0.001 and (****) *p* < 0.0001. Comparative to positive control (LPS) (#) *p* < 0.5, (##) *p* < 0.01, (###) *p* < 0.001 and (####) *p* < 0.0001. Comparative between concentrations (‡) *p* < 0.5, (‡‡) *p* < 0.01, (‡‡‡) *p* < 0.001 and (‡‡‡‡) *p* < 0.0001.

To gain a deeper understanding of HEG’s effects in macrophages, a gene expression analysis of markers associated with inflammatory responses was performed. Consistent with IL-6 release into the supernatant, *IL6* expression was markedly upregulated in response to LPS, with a stronger effect at 24 h. When HEG was added to LPS-stimulated cells, a relative decrease in *IL6* expression was observed compared to the positive control (LPS+) (Figure 4a,b). The expression pattern of *IL10* was consistent with its protein levels in the culture supernatant. In LPS-stimulated cells, 31.25 µg/mL HEG produced slightly lower modulation than 62.50 µg/mL, which exhibited a profile similar to the negative control. Time had no influence on the outcome (Figure 4c,d). Both vascular endothelial growth factor A (*VEGFA*) and transforming growth factor beta (*TGFB1*) were upregulated following LPS treatment (Figure 4e–h). HEG further increased *VEGFA* expression in both unstimulated and LPS-stimulated cells, particularly after 48 h at 62.50 µg/mL (Figure 4e,f). A similar pattern was observed for *TGFB1* (Figure 4g,h). Arginase 2 (*ARG2*) expression was significantly upregulated in response to LPS, with the highest modulation observed at 48 h. At 24 h, the 62.50 µg/mL dose of HEG resulted in the lowest *ARG2* expression. Additionally, at 48 h, both doses of HEG significantly reduced *ARG2* expression, with no significant difference between them (Figure 4i,j).

## 3. Discussion

In recent years, geopropolis, a discarded product of native Brazilian bees, has gained increasing attention for its pharmaceutical potential, warranting further investigation of its biological activities [3]. Accordingly, this study analyzed the composition and anti-inflammatory properties of the hydroalcoholic extract of *Melipona quadrifasciata* geopropolis (HEG).

Geopropolis composition varies with geographic origin, influenced by local flora and climate [3]. Wax content directly reflects regional climatic conditions, as bees incorporate more wax during periods of resin scarcity or when increased hive sealing is required, especially in colder seasons. In the present sample from Ponta Grossa, Paraná (Southern Brazil), wax content reached 51.81%, exceeding the 25% threshold established for raw propolis [26], consistent with the region’s cooler climate, which promotes wax production. The measured water content was 4.52%. Because geopropolis is used for hive sealing, moisture levels vary according to its position within the hive and environmental exposure. Enclosed meliponiculture boxes limit external contact, explaining the observed water content, which complies with the propolis quality standard of a maximum of 8% moisture [26]. Ash content, indicative of mineral residue and resin correlation, was 50.67%. Although the propolis threshold is 5% [26], higher ash levels in geopropolis are expected due to its substantial soil and resin fractions. The composition of both propolis and geopropolis is biome-dependent. Mass spectrometry analysis identified di- and triterpenoids as major constituents (Table 2). This terpenoid-rich composition, likely associated with the coniferous flora of Southern Brazil, including Araucaria and Pinus species, distinguishes our sample from previously reported geopropolis [10,16,17]. Terpenoids are well documented for anti-inflammatory, antimicrobial, and anticancer activities, supporting their pharmacological relevance [27,28]. Within the triterpene class, α and β amyrins and lupeol were identified. These specific compounds are currently being investigated for their ability to inhibit the NF-κB signaling pathway and Toll-like receptor 2 activation—mechanisms critical for mitigating bacterial infection and sepsis—as well as for their antioxidant potential [29,30]. Furthermore, diterpenes such as abietic acid, also detected in our samples, have been previously characterized in the literature for their potent antimicrobial and anti-inflammatory activities [31].

The THP-1 cell line was utilized to investigate the anti-inflammatory effects of HEG. Upon PMA differentiation, THP-1 cells acquire macrophage-like characteristics, making them a robust in vitro model for studying inflammatory processes [32]. Macrophages, central components of the innate immune system, play essential roles in homeostasis through the recognition and phagocytosis of microorganisms, pathogens, and exogenous particles [33]. During infections, activated macrophages release inflammatory mediators including chemokines, cytokines, and proteolytic cascade products. Lipopolysaccharide (LPS), a key constituent of Gram-negative bacterial membranes, is recognized by Toll-like receptor 4 (TLR4) on monocytes and macrophages, initiating a NF-κB, dependent signaling cascade. This process leads to the production of pro-inflammatory cytokines, TNF-α, IL-6, IL-8, and IL-1β, and chemokines such as RANTES (CCL5) and MCP-1 (CCL2) [18,34,35,36].

HEG cytotoxicity was first assessed in THP-1-derived macrophages, confirming that cell viability remained unaffected except at concentrations above 125 µg/mL, where cytotoxicity was observed (Figure 1). Therefore, 31.25 and 62.50 µg/mL were selected for subsequent experiments. In LPS-stimulated macrophages, HEG significantly decreased IL-6 levels, particularly at 62.50 µg/mL (Figure 2a,b). LPS acts as a potent inflammatory stimulus, causing rapid upregulation of IL-10, which helps regulate pro-inflammatory cytokines and maintain immune balance [37,38]. In this study, macrophages treated with 31.25 µg/mL HEG showed increased IL-10 expression, whereas those treated with 62.50 µg/mL showed IL-10 levels near basal detection, indicating improved inflammation control (Figure 2c,d). This reduction in IL-6, together with regulated IL-10 production, suggests a reduced need for anti-inflammatory compensation, resembling the effects observed with dexamethasone. For TNF-α and IL-1β, HEG alone did not modulate cytokine levels. Similarly, HEG treatment did not significantly modulate these cytokines in LPS-stimulated macrophages at 24 h (Figure 2e,g). After 48 h, a slight but statistically significant increase was observed compared with the positive control (LPS+) group (Figure 2f,h). Given the strong pro-inflammatory stimulus induced by LPS, characterized by sharply elevated cytokine release, HEG’s effect on TNF-α and IL-1β was minimal.

Chemokines, cytokines with chemoattractant properties, coordinate immune cell recruitment during inflammation [39,40]. Their expression is mainly induced by pro-inflammatory mediators, including TNF-α and members of the IL-1 family [41]. LPS is a potent inducer of acute inflammation, leading macrophages to produce high levels of MCP-1 (CCL2), RANTES (CCL5), IL-8 (CXCL8), MIG (CXCL9), and CXCL10 via TLR4/NF-κB activation, thereby amplifying the inflammatory response [36,41,42,43,44]. Flow cytometry demonstrated that LPS stimulation upregulated all assessed chemokines compared with negative controls (Figure 3). Notably, HEG treatment significantly reduced CCL2, CCL5, CXCL9, and CXCL10 levels in a dose- and time-dependent manner (Figure 3a–h), suggesting a more controlled inflammatory state, as these chemokines drive monocyte and macrophage recruitment [40]. Such attenuation may decrease pro-inflammatory cytokine secretion, aiding inflammation resolution. No significant changes in CXCL-8 production were observed in the LPS+HEG groups compared with LPS controls, indicating that HEG did not modulate CXCL-8 under these inflammatory conditions. However, macrophages treated with HEG alone exhibited significantly higher CXCL-8 expression compared to negative controls, especially at 62.50 µg/mL (Figure 3i,j). While primarily pro-inflammatory, CXCL-8 is increasingly recognized for its role in tissue repair; it acts as a potent neutrophil chemoattractant and pro-angiogenic factor, facilitating pathogen clearance and supporting endothelial proliferation, migration, and angiogenesis, processes essential for tissue regeneration [45,46,47]. Thus, CXCL-8 contributes to both inflammatory mediation and the proliferative phase of wound healing.

Endotoxin testing further ensured that the observed immunomodulatory effects were not influenced by intrinsic bacterial contamination of the extract.

To further clarify these findings, gene expression analysis was performed for markers involved in inflammation and tissue repair, including *IL1β*, *IL6*, *IL10*, *VEGFA*, *TGFB1*, and *ARG2* (Figure 4). Macrophages were treated under the same conditions as in previous assays. Consistent with cytokine quantification, *IL6* and *IL10* gene expression mirrored protein levels: LPS stimulation caused strong upregulation, while HEG induced dose- and time-dependent downregulation of *IL6*. For *VEGFA* and *TGFB1*, key genes in tissue repair and wound healing, HEG significantly increased expression in a dose- and time-dependent manner, with the strongest responses observed at 48 h and 62.50 µg/mL.

These findings support a pro-regenerative profile at the molecular level, based on the upregulation of pro-angiogenic and tissue repair-associated mediators in unstimulated macrophages.

Additionally, analysis of *ARG2*, a marker of pro-inflammatory M1 macrophages [48,49,50], revealed a significant reduction in expression in HEG-treated cells compared with those exposed only to LPS, most notably after 48 h of treatment.

Altogether, this study demonstrates that the HEG of *M. quadrifasciata*, characterized by a distinctive terpenoid-rich composition, exerts significant immunomodulatory effects in THP-1-derived human macrophages. HEG attenuated LPS-induced pro-inflammatory signaling while modulating mediators associated with resolution and pro-reparative responses. By linking a chemically distinct geopropolis profile to functional effects in a human macrophage model, this work expands current knowledge on the biological potential of Brazilian geopropolis and highlights its relevance as a source of bioactive compounds. These findings provide a foundation for future studies aimed at isolating active fractions and exploring their therapeutic potential in inflammatory and tissue repair contexts.

## 4. Materials and Methods

### 4.1. Geopropolis Sample Collection and Physicochemical Properties

Geopropolis was collected in November of 2022 at the São Miguel Meliponary, located in the city of Ponta Grossa, PR, Brazil (25°05′42″ S, 50°09′43″ W) and stored at −20 °C. The material was kindly provided to Dr. Maria Carolina de Oliveira Ribeiro (Federal University of Technology of Paraná, UTFPR, Ponta Grossa, Paraná, Brazil) for research purposes only. Access to the biological material was registered in the Brazilian National System for the Management of Genetic Heritage and Associated Traditional Knowledge (SISGEN) under access registration number A2F8D4D.

#### 4.1.1. Wax Content

Geopropolis was macerated and homogenized using the quartering method to obtain a representative sample. A portion of 1 g of the material was diluted in 15 mL of a chloroform–acetone solvent (2:1, *v*/*v*) (Merck, Darmstadt, Germany). The solution was manually shaken for 2 min and then allowed to rest for 1 h. Subsequently, it was filtered using qualitative filter paper and incubated at 80 °C for 2 h. The wax content was determined based on the following Equation (1):

% Wax = [(M3 − M2) × 100]/M1 (1)

where M1 is the initial mass of the sample, M2 is the mass of the filter paper, and M3 is the mass of the filter paper with the sample after drying (wax content).

#### 4.1.2. Water Content

The crucible was first dried in a muffle furnace at 550 °C for 30 min. Then, 5 g of the macerated and quartered sample was incubated at 105 °C for 4 h. The water content was determined based on a mass measurement, following Equation (2):

% Water = 100 − [(M3 − M2) × 100]/M1(2)

where M1 is the initial mass of the sample, M2 is the mass of the crucible, and M3 is the mass of the sample and crucible afterwards drying in the muffle.

#### 4.1.3. Ash Content

Following the same initial sample preparation and drying steps, 5 g of the sample (macerated and quartered) was subsequently incinerated for 4 h at 550 °C. The total ash content was determined based on a mass measurement, following Equation (3):

% Ash = [(M3 − M2) × 100]/M1(3)

where M1 is the initial mass of the sample, M2 is the mass of the crucible, and M3 is the mass of the crucible and sample after the muffle.

### 4.2. Preparation of Hydroalcoholic Extract of Geopropolis (HEG)

The hydroalcoholic extract of geopropolis (HEG) was prepared by dissolving 50 g of macerated geopropolis in 100 mL of 70% ethanol (*v*/*v*) (Merck, Darmstadt, Germany). The mixture was subjected to ultrasound for 30 min at 26 °C, followed by filtration through a 45 µM filter. The filtrate was then maintained overnight at 4 °C to allow for the decantation of resins and waxes. After decantation, the extract was centrifuged at 1500 rpm for 10 min at 4 °C to remove insoluble residues. The resulting supernatant was lyophilized and stored at −20 °C.

### 4.3. Bacterial Endotoxin Quantification

To assess the presence of bacterial endotoxin (LPS), a sample of HEG (62.5 µg/mL) diluted in phosphate-buffered saline (PBS) was submitted to the Microbiological Quality Control Sector of the Butantan Institute. The sample was processed according to the sector’s standard operating procedures and analyzed using the Pyrogent Turbidimetric TM-5000 LAL (Limulus Amebocyte Lysate) assay (Kinetic Turbidimetric LAL Assay Test kit, Charles River Laboratories, Wilmington, MA, USA). Results were expressed as endotoxin units per milliliter (EU/mL).

### 4.4. GC-MS Analysis

HEG was analyzed by gas chromatography coupled with mass spectrometry (GC-MS) using a 6850 Network GC System equipped with a 5975C VL MSD detector (Agilent Technologies, Santa Clara, CA, USA) and an HP5-MS capillary column (length 30 m, ID 250 µm, 0.25 µm film thickness) (Agilent Technologies, Santa Clara, CA, USA). The initial column temperature was set to 100 °C for 5 min, and ramped at 5 °C min^−1^ to a final temperature of 320 °C, resulting in a total run time of 57 min. The injection volume was 1 µL, with helium as the carrier gas at a flow rate of 1 mL·min^−1^. The injector, ion source, and quadrupole temperatures were 300 °C, 280 °C, and 180 °C, respectively. MS detection was performed with electron ionization (EI) at 70 eV in full-scan acquisition mode ranging between 50 and 800 *m*/*z* at 2.66 scan s^−1^. Compounds were identified by comparison mass fragmentation patterns with spectra from the NIST digital library spectra 2.0 (2008) and through the evaluation of characteristic fragmentation behavior. n-Alkanes were characterized by comparing retention times with those of a homologous series ranging from C19 to C40.

### 4.5. Monocyte Cultivation and Differentiation into Macrophages

THP-1 cells, a human leukemia-derived monocytic cell line, were acquired from BCRJ (Rio de Janeiro, Brazil). Cells were cultured in 75 cm^2^ flasks at a density of 0.18 to 1.0 × 10^6^ cells/mL (routine culture conditions) in RPMI 1640 medium (Gibco, brand of Thermo Fisher Scientific, Waltham, MA, USA) supplemented with 25 mM glucose, 10 mM HEPES, 1 mM sodium pyruvate, 24 mM sodium bicarbonate, 1% penicillin–streptomycin, and 10% fetal bovine serum (RPMI/10% FBS). The cells were maintained at 37 °C in a humidified incubator with 5% CO_2_. For cytotoxicity and immunomodulatory assays, monocytes were seeded in 96-well plates at 2.5 × 10^4^ cells/well; for RNA extraction, cells were seeded in 24-well plates at 1.65 × 10^5^ cells/well. To induce differentiation into macrophages, RPMI/10% FBS was supplemented with 50 nM phorbol 12-myristate 13-acetate (PMA; Sigma-Aldrich, St. Louis, MO, USA), and cells were incubated at 37 °C with 5% CO_2_ for 48 h. Subsequently, the PMA-containing medium was replaced with fresh RPMI/10% FBS, and cells were cultured for an additional 24 h before use in the assays.

### 4.6. Cell Viability Analysis

Cell viability was analyzed with the (3-(4,5-dimethylthiazol-2-yl)-2,5-diphenyltetrazolium bromide) MTT method. Lyophilized HEG was dissolved in DMSO (Merck, Darmstadt, Germany) at a concentration of 36.60 mg/mL. Following that, a serial dilution was performed using medium culture RPMI/10% FBS at doses ranging from 7.81 to 1000 µg/mL. The cells plated and previously differentiated into macrophages were treated with the different concentrations of HEG solutions and maintained at 37 °C with 5% of CO_2_ for 24 and 48 h. The cells used as controls were maintained either in culture medium alone or in the same volume of DMSO used for sample dilution, at the highest concentration tested. At the end of each time interval, the supernatant was carefully removed, and the adhered cells were incubated at 37 °C with 5% of CO_2_ with MTT (Sigma–Aldrich, St. Louis, MO, USA) solution (0.5 mg/mL) for 3 h. Formazan crystals were dissolved in an equal volume of DMSO (100 µL) for 10 min and the absorbance, optical deviation (OD), was measured at 540 nm in a spectrophotometer (Cytation3, Biotek, Winooski, VT, USA).

### 4.7. Immunomodulatory Assays

To evaluate the immunomodulatory action of HEG, THP-1 cells previously plated and differentiated into macrophages were stimulated with 10 ng/mL of LPS (*Escherichia coli* strain O111:B4, Sigma–Aldrich, St. Louis, MO, USA) for 1 h and posteriorly treated with HEG at final concentrations of 31.25 and 62.50 µg/mL, without the removal of the initial stimulus. In parallel, macrophages were treated with the same concentrations of HEG without LPS stimulation. As controls, cells were maintained in culture medium (negative control), stimulated with LPS alone (10 ng/mL, positive control), or stimulated with LPS for 1 h and then treated with 1 µM dexamethasone (Sigma–Aldrich, St. Louis, MO, USA). Cells were incubated for 24 and 48 h at 37 °C and 5% of CO_2_. At the end of each incubation period, the supernatant was carefully collected, centrifuged at 405× *g* for 10 min at 4 °C to remove nonadherent cells, aliquoted, and stored at −80 °C for subsequent analyses. Cell viability was measured as previously described.

### 4.8. Quantification of Human Inflammatory Cytokines and Chemokines

Human inflammatory cytokines and chemokines (IL-1β, IL-6, IL-10, TNF-α, MCP-1 (CCL2), RANTES (CCL5), IL-8 (CXCL8), MIG (CXCL10) and IP-10 (CXCL9)) in the supernatant of cell culture were analyzed with the Cytometric Bead Array (CBA) method, using the BD™ Human Inflammatory Cytokine Cytometric Bead Array (CBA) kit and BD™ Human Chemokine Cytometric Bead Array (CBA) kit, respectively. Both procedures were made according to the manufacturer’s recommendations (BD Biosciences, San Jose, CA, USA). The acquisition was made with the flow cytometer FACSCanto II, and the concentration of each cytokine was determined using the software FCAP Array 3.0 (BD Biosciences, San Jose, CA, USA).

### 4.9. Gene Expression Analysis by RT-qPCR

To assess gene expression, THP-1 cells previously plated and differentiated into macrophages were stimulated with 10 ng/mL of LPS for 1 h and posteriorly treated with HEG at final concentrations of 31.25 and 62.50 µg/mL. In parallel, macrophages were treated with the same concentrations of HEG without LPS stimulation. As controls, cells were maintained in culture medium (negative control) or stimulated with LPS alone (10 ng/mL, positive control). Cells were incubated for 24 and 48 h at 37 °C and 5% of CO_2_.

After incubation, the culture medium was removed, and total RNA was extracted from cells using the TRIzolTM reagent (Invitrogen, Thermo Fisher Scientific Inc., Waltham, MA, USA) according to the manufacturer’s recommendations. The RNA concentration was measured at 260 nm (1 D.O. = 40 µg RNA/mL), and purity was assessed using a 260/280 nm ratio (≥1.8 considered pure). Total RNA was first treated with DNase I (Invitrogen, Thermo Fisher Scientific Inc., Waltham, MA, USA) to remove contaminating genomic DNA. Reverse transcription to complementary DNA (cDNA) was performed using 1 µg of DNase-treated RNA using the SuperScriptTM III Reverse Transcriptase kit (Invitrogen, Thermo Fisher Scientific Inc., Waltham, MA, USA) according to the manufacturer’s instructions.

Relative mRNA expression of target genes was quantified by qPCR. Reactions were performed with 10-fold diluted cDNA from each biological sample and 500 nM of each primer (forward and reverse) with Fast SYBRTM Green Master Mix (Applied Biosystems, Thermo Fisher Scientific Inc., Waltham, MA, USA) on a QuantStudioTM 3 Real-Time PCR System (Applied Biosystems, Thermo Fisher Scientific Inc., Waltham, MA, USA). *Glyceraldehyde-3-phosphate dehydrogenase* (*GAPDH*) and *Ribosomal protein L13A* (*RPL13A*) were used as endogenous reference genes. Results are expressed as relative changes in gene expression in treated cells versus control cells, normalized to the geometric mean of the Ct values of the two reference genes, using the 2^−∆∆CT^ method [51]. A complete list of primers is provided in Supplementary Table S1.

### 4.10. Statistical Analysis

Statistical analyses were performed using GraphPad Prism version 8.00 (GraphPad Software, Inc, Boston, MA, USA). Data normality was assessed using the Shapiro–Wilk test. For parametric data, differences between groups and across time points were evaluated by one-way ANOVA, followed by Dunnett’s multiple comparison test against the control group. Results are presented as the mean ± standard error of the mean (SEM) from a minimum of two independent experiments, each performed in triplicate. Statistical significance was defined as *p* < 0.05.

## Figures and Tables

**Table 1:** Physicochemical properties of HEG.

Parameters	Mean in % (±SEM)
Wax	51.81 (±11.06)
Water content	4.52 (±0.4957)
Ash	50.67 (±1.559)

**Table 2:** Identification of compounds by GC-MS found in HEG.

	Compounds	% Relative
Diterpenoids		
1	Manool	1.63
2	Ferruginol	2.47
3	15-oxo-labd-8(17)-en-18-oic acid	1.25
4	Sandaracopimaric acid	0.80
5	Pimaric acid	3.99
6	Communic acid	9.85
7	Isopimaric acid	2.34
8	Sclareol	4.47
9	Dehydroabietic acid	2.14
10	Epi-Manool	7.91
11	Abietic acid	2.72
12	15-Hydroxylabd-8(17).13(*Z*)-dien-18-oic acid	9.70
13	15-Hydroxylabd-8(17).13(*Z*)-dien-18-al	1.43
14	7α-Hydroxydehydroabietic acid	0.87
15	15-Hydroxylabd-8(17)-en-18-oic acid	2.72
16	15-Hydroxylabd-8(17).13(*E*)-dien-18-oic acid	26.17
17	15-Hydroxylabd-8(17).13(*E*)-dien-18-al	0.98
18	Agathic acid 15-methyl-ester	1.02
19	Agathic acid	0.91
Triterpenoids		
20	α-Amyrin	1.14
21	β-Amyrin	2.37
22	Lupeol	0.53
Fatty acid derivatives		
23	Azelaic acid	0.39
24	Palmitic acid	0.47
25	Glyceryl stearate	0.85
Sugars		
26	Galactose	0.48
Identified compounds		
	Diterpenoids	83.37
	Triterpenoids	4.04
	Fatty acid derivatives	1.71
	Sugars	0.48
Non-identified		10.40

## Data Availability

The original contributions presented in this study are included in the article/Supplementary Materials. Further inquiries can be directed to the corresponding author.

## Supplementary Materials

The following supporting information can be downloaded at: https://www.mdpi.com/article/10.3390/ijms27073229/s1.

## Abbreviations

The following abbreviations are used in this manuscript:

LPS	Lipopolysaccharide
CCL5	C-C Motif Chemokine Ligand 5
TGFB1	Transforming Growth Factor Beta 1
ARG2	Arginase 2
CBA	Cytometric Bead Array
CCL2	C-C Motif Chemokine Ligand 2
CXCL10	C-X-C Motif Chemokine Ligand 10
CXCL-8	C-X-C Motif Chemokine Ligand 8 (also known as IL-8)
CXCL9	C-X-C Motif Chemokine Ligand 9
DMSO	Dimethyl Sulfoxide
ELISA	Enzyme-Linked Immunosorbent Assay
FBS	Fetal Bovine Serum
GAPDH	Glyceraldehyde-3-Phosphate Dehydrogenase
GC-MS	Gas Chromatography–Mass Spectrometry
HEG	Hydroalcoholic Extract of Geopropolis
IL-10	Interleukin-10
IL-1β	Interleukin-1 Beta
IL-6	Interleukin-6
M1	Classically Activated (Pro-inflammatory) Macrophage Phenotype
M2	Alternatively Activated (Anti-inflammatory/Regulatory) Macrophage Phenotype
MTT	3-(4,5-Dimethylthiazol-2-yl)-2,5-Diphenyltetrazolium Bromide
NF-κB	Nuclear Factor Kappa B
PMA	Phorbol 12-Myristate 13-Acetate
RPL13A	Ribosomal Protein L13a
RT-qPCR	Reverse Transcription Quantitative Polymerase Chain Reaction
THP-1	Human Monocytic Cell Line (derived from acute monocytic leukemia)
TLR4	Toll-Like Receptor 4
TNF-α	Tumor Necrosis Factor Alpha
