# Supplementary material for: Melipona quadrifasciata Geopropolis Extract as a Modulator of Inflammation and Pro-Regenerative Responses in Human Macrophages
Source: Int J Mol Sci. 2026 Apr 2;27(7):3229. doi: 10.3390/ijms27073229 (PMC13072738; doi:10.3390/ijms27073229)
Supplement: Supplementary file 1 [file ijms-27-03229-s001.zip › ijms-4135829-supplementary.pdf]

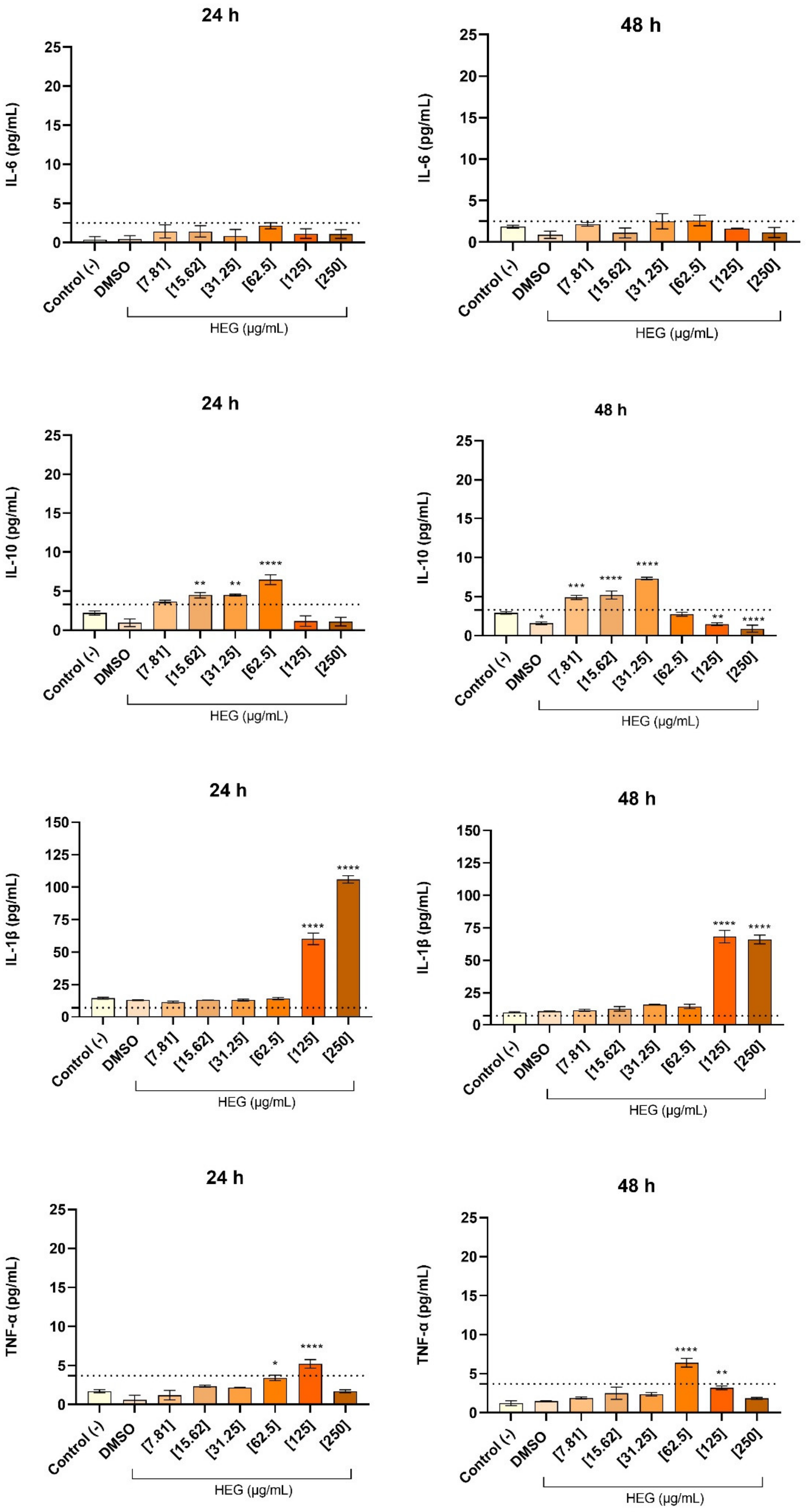
24 h
48 h
IL-6 (pg/mL)
IL-10 (pg/mL)
IL-1β (pg/mL)
TNF-α (pg/mL)
Control (-)
DMSO
[7.81]
[15.62]
[31.25]
[62.5]
[125]
[250]
HEG (µg/mL)
0
5
10
15
20
25
50
75
100
125
150
*
**
***
****

Figure S1. Cytokine levels of macrophages treated with increasing doses of HEG. THP-1 differentiated macrophages (2.5 x 10^{4} cells/well) were stimulated with doses ranging from 7.81 to 1,000 μg/mL of HEG. Cells not treated with HEG or with the highest concentration of vehicle DMSO were used as controls. After 24 and 48 hours of incubation, the production of cytokines in the supernatants of the cultures was determined by CBA. Statistical analysis was performed by One-way ANOVA, followed by Dunnett's post-test compared to the negative control. (*) $p<0.5$, (**) $p<0.01$, (***) $p<0.001$ and (****) $p<0.0001$. The dotted line represents the limit of detection for each analyte.

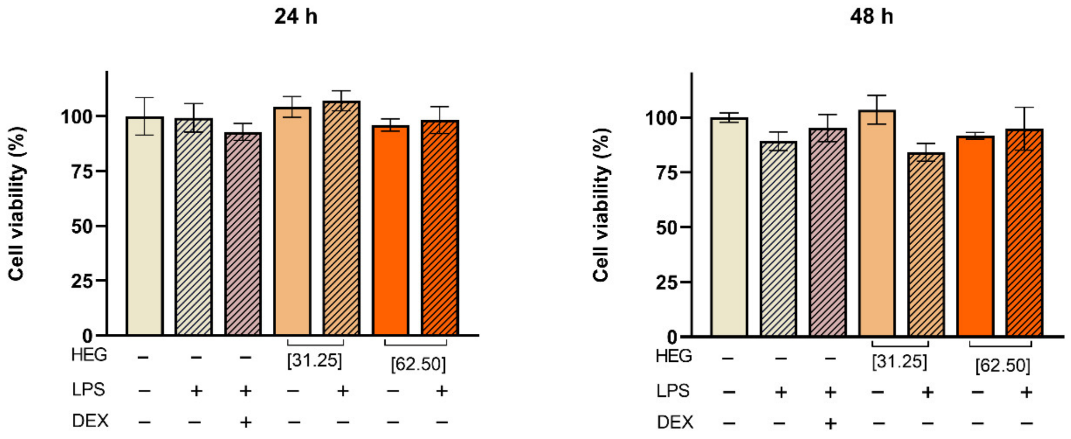

Figure S2. Cytotoxic effects of HEG on macrophages after stimulation with LPS. THP-1 differentiated macrophages (2.5 x 10^{4} cells/well) were stimulated or not with LPS (10 ng/mL) for 1 hour, then treated with HEG at 31.25 and 62.5 μg/mL. As controls, cells were maintained in culture medium (negative control), stimulated with LPS alone (positive control) or stimulated with LPS for 1 hour followed by treatment with dexamethasone (1 μM). After 24 and 48 hours of incubation, cells viability was assessed by MTT assay. The results represent two separate experiments performed in triplicate and are expressed as the mean ± SEM. Statistical analysis was performed by One-way ANOVA, followed by Dunnett's post-test comparative to the negative control.

Table S1. List of primers.

Gene	Encoded protein	GenBank	Oligonucleotide sequence
IL6 (human)	Interleukin 6	NM_000600.5	F: GTACATCCTCGACGGCATCTC
			R: TCACCAGGCAAGTCTCCTCAT
IL10 (human)	Interleukin 10	NM_000572.3	F: GCTGAGAACCAAGACCCAGACA
			R: TAAAGGCATTCTTCACCTGCTCC
VEGFA (human)	Vascular Endothelial Growth Factor A	NM_001025366.3	F: CCCACTGAGGAGTCCAACATC
			R: CTGCATTCACATTTGTTGTGCTG
TGFB1 (human)	Transforming Growth Factor Beta	NM_000660.7	F: CGCGTGCTAATGGTGGAAAC
			R: GTTCAGGTACCGCTTCTCGG
ARG2 (human)	Arginase 2	NM_001172.4	F: ACTCCGTGGCTGTGATAGGAG
			R: AGCCCAAACTGGAGAGCCTT
GAPDH (human)	Glyceraldehyde 3-phosphate dehydrogenase	NM_002046.7	F: GTGAAGGTCGGAGTCAACGG
			R: AGTTGAGGTCAATGAAGGGGTC
RPL13A (human)	Ribosomal Protein L13a	NM_012423	F: GTATGCTGCCCCACAAAACC
			R: CTTCAGACGCACGACCTTGA
